# Supplementary material for: Neuroendocrine Biomarkers of Herbal Medicine for Major Depressive Disorder: A Systematic Review and Meta-Analysis
Source: Pharmaceuticals (Basel). 2023 Aug 18;16(8):1176. doi: 10.3390/ph16081176 (PMC10458856; doi:10.3390/ph16081176)
Supplement: Supplementary file 1 [file pharmaceuticals-16-01176-s001.zip › Supplementary Figure S1-6. Forest plot for comparison of HAMD score and adverse effective rate.pdf]

## Supplementary Figure S1-6. Forest plot for comparison of HAMD score and adverse effective rate.

| Study or Subgroup | HM Mean | HM SD | HM Total | AD Mean | AD SD | AD Total | Weight | Std. Mean Difference IV, Random, 95% CI |
|---|---|---|---|---|---|---|---|---|
| Li 2007 | 11.22 | 8.82 | 32 | 8.77 | 7.62 | 31 | 25.1% | 0.29 [−0.20, 0.79] |
| Liang 2012 | 10.47 | 4.38 | 30 | 10.18 | 3.75 | 30 | 25.0% | 0.07 [−0.44, 0.58] |
| Wang 2005 | 7.72 | 2.52 | 32 | 9.19 | 3.21 | 31 | 25.0% | −0.50 [−1.01, −0.00] |
| Zhou 2015 | 11.19 | 1.45 | 40 | 13.87 | 1.8 | 40 | 25.0% | −1.62 [−2.13, −1.12] |
| **Total (95% CI)** | | | **134** | | | **132** | **100.0%** | **−0.44 [−1.28, 0.40]** |

Heterogeneity: $Tau^2 = 0.66$; $Chi^2 = 33.15$, df = 3 (P < 0.00001); $I^2 = 91\%$
Test for overall effect: Z = 1.03 (P = 0.30)

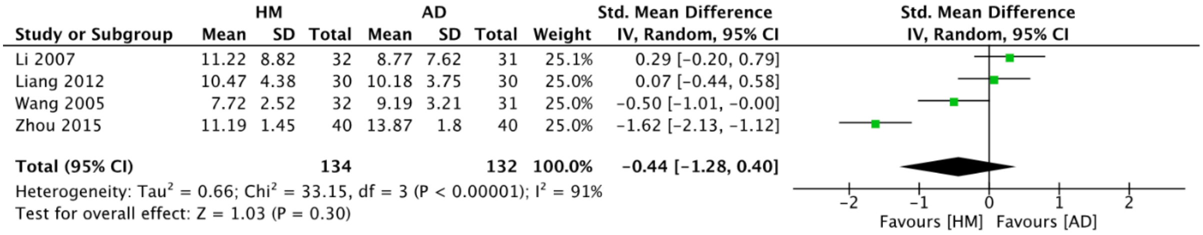


**Supplementary Figure S1.** Forest plot of the comparison between herbal medicine versus antidepressant assessing HAMD. AD, antidepressant; HAMD, Hamilton Depression Scale; HM, herbal medicine.

| Study or Subgroup | HM Mean | HM SD | HM Total | AD Mean | AD SD | AD Total | Weight | Std. Mean Difference IV, Random, 95% CI |
|---|---|---|---|---|---|---|---|---|
| **2.2.1 SSRI** | | | | | | | | |
| Liang 2012 | 10.47 | 4.38 | 30 | 10.18 | 3.75 | 30 | 25.0% | 0.07 [−0.44, 0.58] |
| Wang 2005 | 7.72 | 2.52 | 32 | 9.19 | 3.21 | 31 | 25.0% | −0.50 [−1.01, −0.00] |
| Zhou 2015 | 11.19 | 1.45 | 40 | 13.87 | 1.8 | 40 | 25.0% | −1.62 [−2.13, −1.12] |
| **Subtotal (95% CI)** | | | **102** | | | **101** | **74.9%** | **−0.69 [−1.66, 0.29]** |
| Heterogeneity: $Tau^2 = 0.67$; $Chi^2 = 22.14$, df = 2 (P < 0.0001); $I^2 = 91\%$ | | | | | | | | |
| Test for overall effect: Z = 1.38 (P = 0.17) | | | | | | | | |
| **2.2.2 TCAs** | | | | | | | | |
| Li 2007 | 11.22 | 8.82 | 32 | 8.77 | 7.62 | 31 | 25.1% | 0.29 [−0.20, 0.79] |
| **Subtotal (95% CI)** | | | **32** | | | **31** | **25.1%** | **0.29 [−0.20, 0.79]** |
| Heterogeneity: Not applicable | | | | | | | | |
| Test for overall effect: Z = 1.16 (P = 0.25) | | | | | | | | |
| **Total (95% CI)** | | | **134** | | | **132** | **100.0%** | **−0.44 [−1.28, 0.40]** |

Heterogeneity: $Tau^2 = 0.66$; $Chi^2 = 33.15$, df = 3 (P < 0.00001); $I^2 = 91\%$
Test for overall effect: Z = 1.03 (P = 0.30)
Test for subgroup differences: $Chi^2 = 3.09$, df = 1 (P = 0.08), $I^2 = 67.7\%$

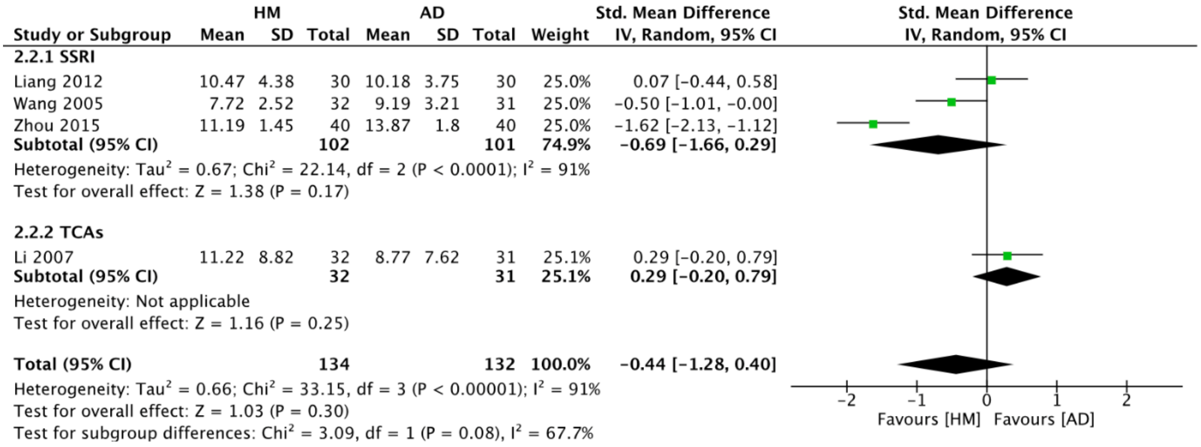


**Supplementary Figure S2.** Forest plot of the comparison between herbal medicine versus antidepressant assessing HAMD, Subgroup analysis according to type of AD. AD, antidepressant; HAMD, Hamilton Depression Scale; HM, herbal medicine.

| Study or Subgroup | HM+AD Mean | HM+AD SD | HM+AD Total | AD Mean | AD SD | AD Total | Weight | Std. Mean Difference IV, Random, 95% CI |
|---|---|---|---|---|---|---|---|---|
| **2.3.1 <4weeks** | | | | | | | | |
| Qin 2020 | 7.99 | 1.02 | 46 | 13.96 | 1.99 | 46 | 4.4% | −3.74 [−4.43, −3.06] |
| Zhu 2016 | 16 | 6.5 | 13 | 23.22 | 6.03 | 9 | 4.0% | −1.10 [−2.02, −0.18] |
| **Subtotal (95% CI)** | | | **59** | | | **55** | **8.4%** | **−2.44 [−5.03, 0.15]** |
| Heterogeneity: Tau² = 3.32; Chi² = 20.29, df = 1 (P < 0.00001); I² = 95% | | | | | | | | |
| Test for overall effect: Z = 1.85 (P = 0.06) | | | | | | | | |
| **2.3.2 4~8weeks** | | | | | | | | |
| Du 2018 | 7.79 | 2.46 | 78 | 13.34 | 2.74 | 78 | 4.7% | −2.12 [−2.52, −1.73] |
| Gong 2016 | 5.7 | 1.6 | 70 | 7.2 | 1.8 | 70 | 4.8% | −0.88 [−1.22, −0.53] |
| Huo 2013 | 13.2 | 2.16 | 43 | 15.7 | 2.28 | 42 | 4.7% | −1.12 [−1.57, −0.66] |
| Li 2016 | 4.68 | 3.72 | 40 | 9.32 | 3.91 | 40 | 4.6% | −1.20 [−1.68, −0.73] |
| Li 2017 | 12.87 | 4.99 | 30 | 16.97 | 5.68 | 30 | 4.6% | −0.76 [−1.28, −0.23] |
| Liu 2017 | 12.63 | 3.41 | 51 | 17.58 | 4.17 | 51 | 4.7% | −1.29 [−1.72, −0.86] |
| Sun 2020 | 8.17 | 3.24 | 48 | 14.39 | 4.83 | 48 | 4.7% | −1.50 [−1.96, −1.05] |
| Tan 2020 | 13.12 | 2.69 | 43 | 17.32 | 3.74 | 43 | 4.6% | −1.28 [−1.74, −0.81] |
| Tong 2016 | 7.75 | 2.07 | 30 | 9.16 | 2.12 | 30 | 4.6% | −0.66 [−1.19, −0.14] |
| Tong 2018 | 6.1 | 1.8 | 58 | 8.6 | 2.2 | 58 | 4.7% | −1.24 [−1.63, −0.84] |
| Wang 2017 | 11.73 | 1.56 | 100 | 16.58 | 2.15 | 100 | 4.7% | −2.57 [−2.95, −2.20] |
| Wang 2018 | 15.37 | 1.62 | 66 | 22.73 | 2.46 | 65 | 4.5% | −3.52 [−4.07, −2.97] |
| Wang 2020 | 7.24 | 3.43 | 46 | 9.61 | 3.82 | 46 | 4.7% | −0.65 [−1.07, −0.23] |
| Wang 2022 | 13.56 | 2.18 | 41 | 17.02 | 2.24 | 41 | 4.6% | −1.55 [−2.05, −1.05] |
| Wu 2019a | 16.48 | 1.63 | 40 | 26.03 | 1.46 | 40 | 3.8% | −6.11 [−7.18, −5.05] |
| Wu 2019b | 17.48 | 1.73 | 40 | 25.03 | 2.46 | 40 | 4.3% | −3.52 [−4.23, −2.81] |
| Wu 2020 | 17.48 | 1.73 | 40 | 25.03 | 2.46 | 40 | 4.3% | −3.52 [−4.23, −2.81] |
| Zhu 2020 | 9.43 | 4.71 | 42 | 13.93 | 7.39 | 41 | 4.7% | −0.72 [−1.17, −0.28] |
| **Subtotal (95% CI)** | | | **906** | | | **903** | **82.3%** | **−1.84 [−2.30, −1.38]** |
| Heterogeneity: Tau² = 0.92; Chi² = 290.68, df = 17 (P < 0.00001); I² = 94% | | | | | | | | |
| Test for overall effect: Z = 7.81 (P < 0.00001) | | | | | | | | |
| **2.3.3 12weeks** | | | | | | | | |
| Cheng 2018 | 6.91 | 1.04 | 50 | 7.58 | 1.74 | 50 | 4.7% | −0.46 [−0.86, −0.07] |
| Guo 2015 | 13.19 | 2.11 | 49 | 16.28 | 2.29 | 49 | 4.7% | −1.39 [−1.84, −0.95] |
| **Subtotal (95% CI)** | | | **99** | | | **99** | **9.4%** | **−0.92 [−1.83, −0.01]** |
| Heterogeneity: Tau² = 0.38; Chi² = 9.35, df = 1 (P = 0.002); I² = 89% | | | | | | | | |
| Test for overall effect: Z = 1.99 (P = 0.05) | | | | | | | | |
| **Total (95% CI)** | | | **1064** | | | **1057** | **100.0%** | **−1.81 [−2.23, −1.38]** |
| Heterogeneity: Tau² = 0.96; Chi² = 359.29, df = 21 (P < 0.00001); I² = 94% | | | | | | | | |
| Test for overall effect: Z = 8.32 (P < 0.00001) | | | | | | | | |
| Test for subgroup differences: Chi² = 3.44, df = 2 (P = 0.18), I² = 41.8% | | | | | | | | |

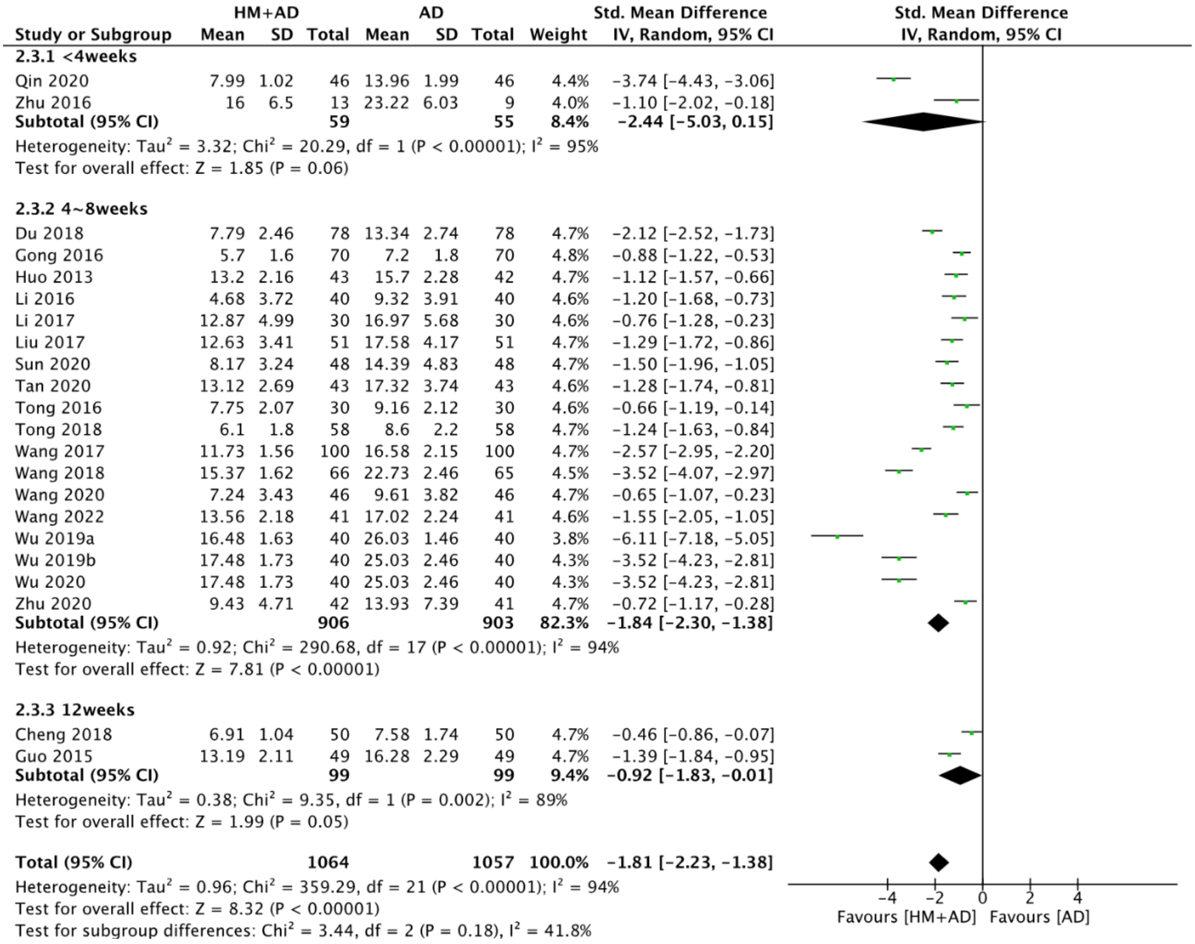


**Supplementary Figure S3.** Forest plot of the comparison between herbal medicine plus antidepressant versus antidepressant alone assessing HAMD, Subgroup analysis according to duration of treatment. AD, antidepressant; HAMD, Hamilton Depression Scale; HM, herbal medicine.

| Study or Subgroup | HM+AD Mean | HM+AD SD | HM+AD Total | AD Mean | AD SD | AD Total | Weight | Std. Mean Difference IV, Random, 95% CI |
|---|---|---|---|---|---|---|---|---|
| **2.4.1 SSRI** | | | | | | | | |
| Du 2018 | 7.79 | 2.46 | 78 | 13.34 | 2.74 | 78 | 4.7% | −2.12 [−2.52, −1.73] |
| Guo 2015 | 13.19 | 2.11 | 49 | 16.28 | 2.29 | 49 | 4.7% | −1.39 [−1.84, −0.95] |
| Huo 2013 | 13.2 | 2.16 | 43 | 15.7 | 2.28 | 42 | 4.7% | −1.12 [−1.57, −0.66] |
| Li 2017 | 12.87 | 4.99 | 30 | 16.97 | 5.68 | 30 | 4.6% | −0.76 [−1.28, −0.23] |
| Qin 2020 | 7.99 | 1.02 | 46 | 13.96 | 1.99 | 46 | 4.4% | −3.74 [−4.43, −3.06] |
| Sun 2020 | 8.17 | 3.24 | 48 | 14.39 | 4.83 | 48 | 4.7% | −1.50 [−1.96, −1.05] |
| Tong 2016 | 7.75 | 2.07 | 30 | 9.16 | 2.12 | 30 | 4.6% | −0.66 [−1.19, −0.14] |
| Tong 2018 | 6.1 | 1.8 | 58 | 8.6 | 2.2 | 58 | 4.7% | −1.24 [−1.63, −0.84] |
| Wang 2022 | 13.56 | 2.18 | 41 | 17.02 | 2.24 | 41 | 4.6% | −1.55 [−2.05, −1.05] |
| Wu 2019a | 16.48 | 1.63 | 40 | 26.03 | 1.46 | 40 | 3.8% | −6.11 [−7.18, −5.05] |
| Wu 2020 | 17.48 | 1.73 | 40 | 25.03 | 2.46 | 40 | 4.3% | −3.52 [−4.23, −2.81] |
| Zhu 2016 | 16 | 6.5 | 13 | 23.22 | 6.03 | 9 | 4.0% | −1.10 [−2.02, −0.18] |
| Zhu 2020 | 9.43 | 4.71 | 42 | 13.93 | 7.39 | 41 | 4.7% | −0.72 [−1.17, −0.28] |
| **Subtotal (95% CI)** | | | **558** | | | **552** | **58.3%** | **−1.90 [−2.47, −1.33]** |
| Heterogeneity: $Tau^2 = 1.01$; $Chi^2 = 187.53$, df = 12 (P < 0.00001); $I^2 = 94\%$ | | | | | | | | |
| Test for overall effect: Z = 6.52 (P < 0.00001) | | | | | | | | |
| **2.4.2 SNRI** | | | | | | | | |
| Cheng 2018 | 6.91 | 1.04 | 50 | 7.58 | 1.74 | 50 | 4.7% | −0.46 [−0.86, −0.07] |
| Gong 2016 | 5.7 | 1.6 | 70 | 7.2 | 1.8 | 70 | 4.8% | −0.88 [−1.22, −0.53] |
| Liu 2017 | 12.63 | 3.41 | 51 | 17.58 | 4.17 | 51 | 4.7% | −1.29 [−1.72, −0.86] |
| Wang 2017 | 11.73 | 1.56 | 100 | 16.58 | 2.15 | 100 | 4.7% | −2.57 [−2.95, −2.20] |
| Wang 2018 | 15.37 | 1.62 | 66 | 22.73 | 2.46 | 65 | 4.5% | −3.52 [−4.07, −2.97] |
| **Subtotal (95% CI)** | | | **337** | | | **336** | **23.4%** | **−1.73 [−2.75, −0.72]** |
| Heterogeneity: $Tau^2 = 1.28$; $Chi^2 = 121.81$, df = 4 (P < 0.00001); $I^2 = 97\%$ | | | | | | | | |
| Test for overall effect: Z = 3.36 (P = 0.0008) | | | | | | | | |
| **2.4.4 Atypical** | | | | | | | | |
| Li 2016 | 4.68 | 3.72 | 40 | 9.32 | 3.91 | 40 | 4.6% | −1.20 [−1.68, −0.73] |
| Wang 2020 | 7.24 | 3.43 | 46 | 9.61 | 3.82 | 46 | 4.7% | −0.65 [−1.07, −0.23] |
| Wu 2019b | 17.48 | 1.73 | 40 | 25.03 | 2.46 | 40 | 4.3% | −3.52 [−4.23, −2.81] |
| **Subtotal (95% CI)** | | | **126** | | | **126** | **13.6%** | **−1.76 [−3.21, −0.31]** |
| Heterogeneity: $Tau^2 = 1.57$; $Chi^2 = 46.83$, df = 2 (P < 0.00001); $I^2 = 96\%$ | | | | | | | | |
| Test for overall effect: Z = 2.38 (P = 0.02) | | | | | | | | |
| **2.4.5 TCAs** | | | | | | | | |
| Tan 2020 | 13.12 | 2.69 | 43 | 17.32 | 3.74 | 43 | 4.6% | −1.28 [−1.74, −0.81] |
| **Subtotal (95% CI)** | | | **43** | | | **43** | **4.6%** | **−1.28 [−1.74, −0.81]** |
| Heterogeneity: Not applicable | | | | | | | | |
| Test for overall effect: Z = 5.38 (P < 0.00001) | | | | | | | | |
| **Total (95% CI)** | | | **1064** | | | **1057** | **100.0%** | **−1.81 [−2.23, −1.38]** |
| Heterogeneity: $Tau^2 = 0.96$; $Chi^2 = 359.29$, df = 21 (P < 0.00001); $I^2 = 94\%$ | | | | | | | | |
| Test for overall effect: Z = 8.32 (P < 0.00001) | | | | | | | | |
| Test for subgroup differences: $Chi^2 = 2.94$, df = 3 (P = 0.40), $I^2 = 0\%$ | | | | | | | | |

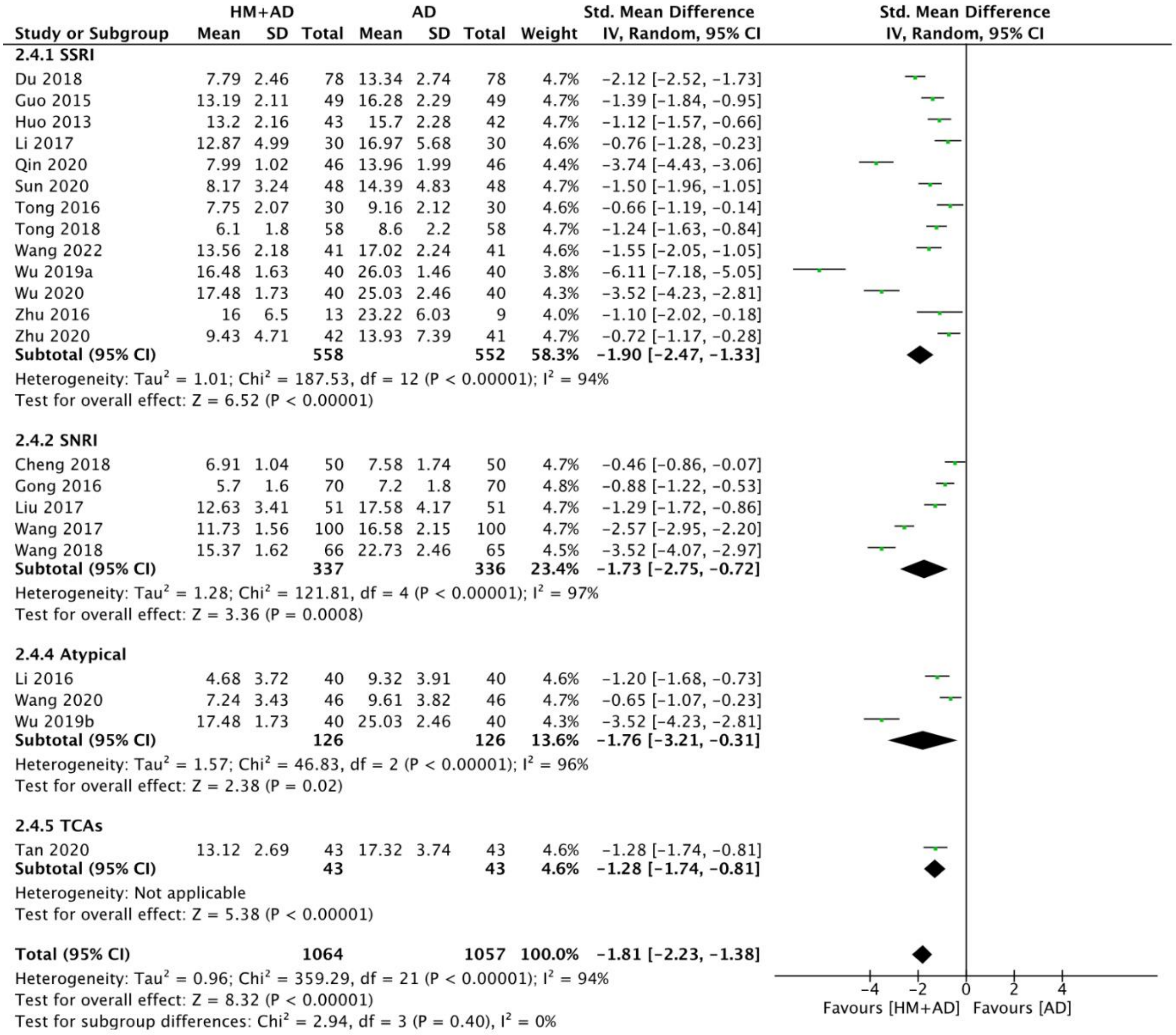


**Supplementary Figure S4.** Forest plot of the comparison between herbal medicine plus antidepressant versus antidepressant alone assessing HAMD, Subgroup analysis according to type of AD. AD, antidepressant; HAMD, Hamilton Depression Scale; HM, herbal medicine.

| Study or Subgroup | HM Events | HM Total | AD Events | AD Total | Weight | Risk Ratio M-H, Random, 95% CI |
|---|---|---|---|---|---|---|
| Liang 2012 | 1 | 30 | 30 | 30 | 47.6% | 0.05 [0.01, 0.23] |
| Su 2020 | 6 | 67 | 9 | 67 | 52.4% | 0.67 [0.25, 1.77] |
| **Total (95% CI)** | | **97** | | **97** | **100.0%** | **0.19 [0.01, 3.15]** |
| Total events | 7 | | 39 | | | |

Heterogeneity: $Tau^2 = 3.64$; $Chi^2 = 9.24$, df = 1 (P = 0.002); $I^2 = 89\%$
Test for overall effect: Z = 1.15 (P = 0.25)

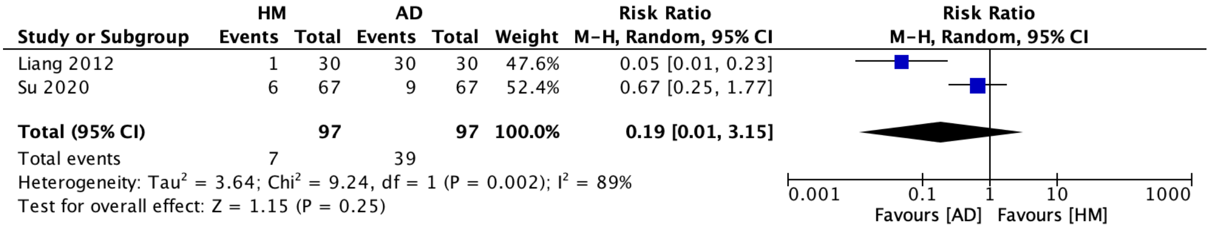


**Supplementary Figure S5.** Forest plot of the comparison between herbal medicine versus antidepressant assessing adverse effective rate.

AD, antidepressant; HM, herbal medicine.

| Study or Subgroup | HM+AD Events | HM+AD Total | AD Events | AD Total | Weight | Risk Ratio M-H, Random, 95% CI |
|---|---|---|---|---|---|---|
| Chen 2021 | 13 | 150 | 10 | 150 | 9.5% | 1.30 [0.59, 2.87] |
| Du 2018 | 13 | 78 | 22 | 78 | 15.9% | 0.59 [0.32, 1.09] |
| Li 2017 | 2 | 30 | 5 | 30 | 2.5% | 0.40 [0.08, 1.90] |
| Liu 2021 | 5 | 48 | 13 | 48 | 6.7% | 0.38 [0.15, 1.00] |
| Pan 2018 | 6 | 50 | 14 | 50 | 7.9% | 0.43 [0.18, 1.03] |
| Sun 2020 | 6 | 48 | 10 | 48 | 7.0% | 0.60 [0.24, 1.52] |
| Tong 2016 | 7 | 30 | 12 | 30 | 9.8% | 0.58 [0.27, 1.28] |
| Wang 2017 | 10 | 100 | 36 | 100 | 14.4% | 0.28 [0.15, 0.53] |
| Wang 2018 | 2 | 66 | 9 | 65 | 2.7% | 0.22 [0.05, 0.97] |
| Wu 2019a | 3 | 40 | 7 | 40 | 3.7% | 0.43 [0.12, 1.54] |
| Wu 2019b | 5 | 40 | 10 | 40 | 6.3% | 0.50 [0.19, 1.33] |
| Wu 2020 | 5 | 40 | 10 | 40 | 6.3% | 0.50 [0.19, 1.33] |
| Zhu 2020 | 5 | 42 | 16 | 41 | 7.3% | 0.31 [0.12, 0.76] |
| **Total (95% CI)** | | **762** | | **760** | **100.0%** | **0.48 [0.38, 0.62]** |
| Total events | 82 | | 174 | | | |

Heterogeneity: $Tau^2 = 0.00$; $Chi^2 = 12.18$, df = 12 (P = 0.43); $I^2 = 1\%$
Test for overall effect: Z = 5.80 (P < 0.00001)

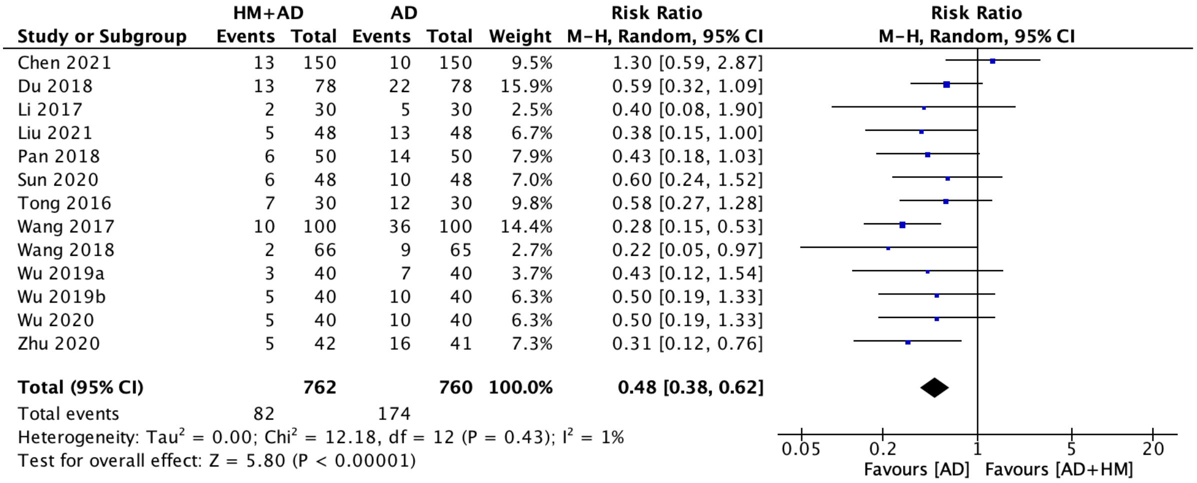


**Supplementary Figure S6.** Forest plot of the comparison between herbal medicine plus antidepressant versus antidepressant alone assessing adverse effective rate. AD, antidepressant; HM, herbal medicine.
