# Supplementary material for: Neuroendocrine Biomarkers of Herbal Medicine for Major Depressive Disorder: A Systematic Review and Meta-Analysis
Source: Pharmaceuticals (Basel). 2023 Aug 18;16(8):1176. doi: 10.3390/ph16081176 (PMC10458856; doi:10.3390/ph16081176)
Supplement: Supplementary file 1 [file pharmaceuticals-16-01176-s001.zip › Supplementary File S1. Search terms used in each database-proof.pdf]

Supplementary File S1. Search terms used in each database

Medline via PubMed

	Searches	Results
#1	"Depressive Disorder"[MeSH] OR "Depression"[MeSH] OR "Depression"[Title/abstract] OR "Depressive Disorder"[Title/abstract] OR depressive[Title/abstract] OR depress*[Title/abstract] OR dysthymia[Title/abstract]	579,899
#2	"Plants, Medicinal"[MeSH] OR "Drugs, Chinese Herbal"[MeSH] OR "Medicine, Chinese Traditional"[MeSH] OR "Medicine, Kampo"[MeSH] OR "Medicine, Korean Traditional"[MeSH] OR "Herbal Medicine"[MeSH] OR "Prescription Drugs"[MeSH] OR "traditional Korean medicine"[Title/abstract] OR "traditional Chinese medicine"[Title/abstract] OR "Traditional oriental medicine"[Title/abstract] OR "Kampo medicine"[Title/abstract] OR "alternative medicine"[Title/abstract] OR "complementary medicine"[Title/abstract] OR herb*[Title/abstract] OR decoction*[Title/abstract] OR botanic*[Title/abstract]	266,313
#3	randomized controlled trial[Publication Type] OR controlled clinical trial[Publication Type] OR randomized[Title/abstract] OR placebo[Title/abstract] OR randomly[Title/abstract] OR trial[Title/abstract] OR groups[Title/abstract]	3,548,743
#4	neuroscience[MeSH] OR "endocrine system"[MeSH] OR serotonin[MeSH] OR "Adrenal Cortex Hormones"[MeSH] OR "Brain-Derived Neurotrophic Factor"[MeSH] OR "Transforming Growth Factor beta"[MeSH] OR Norepinephrine[MeSH] OR Dopamine[MeSH] OR "nerve growth factor"[MeSH] OR "neurological pathway"[Title/abstract] OR neuroanatom*[Title/abstract] OR neurotransmitter[Title/abstract] OR "neurotropic factor"[Title/abstract] OR "scientific basis"[Title/abstract] OR "5-HT"[Title/abstract] OR ACTH[Title/abstract] OR BDNF[Title/abstract] OR "HPA axis"[Title/abstract] OR monoamine[Title/abstract]	1,140,911
#5	#1 AND #2 AND #3 AND #4	177

EMBASE via Elsevier

	Searches	Results
#1	'depression'/exp OR 'depression':ab,ti OR 'depression disorder':ab,ti OR 'depressive':ab,ti OR depress*:ab,ti OR dysthymia:ab,ti	915,519
#2	'medicinal plant'/exp OR 'medicinal plant' OR 'herbaceous agent'/exp OR 'herbaceous agent':ab,ti OR 'chinese medicine'/exp OR 'chinese medicine':ab,ti OR 'kampo medicine'/exp OR 'kampo medicine':ab,ti OR ('kampo medicine'/exp AND 'drug'/exp) OR 'kampo medicine (drug)':ab,ti OR 'korean medicine'/exp OR 'korean medicine':ab,ti OR 'herbal medicine'/exp OR 'herbal medicine':ab,ti OR 'prescription drug'/exp OR 'prescription drug':ab,ti OR 'oriental medicine'/exp OR 'oriental medicine':ab,ti OR 'alternative medicine'/exp OR 'alternative medicine':ab,ti OR 'complementary medicine':ab,ti OR 'herb'/exp OR 'herb':ab,ti OR 'decoction*':ab,ti OR 'botanic*':ab,ti	513,264
#3	'randomized controlled trial'/de OR 'controlled clinical trial'/de OR randomized:ab,ti OR placebo:ab,ti OR randomly:ab,ti OR trial:ab,ti OR groups:ti	2,317,192
#4	'neuroscience'/exp OR 'neurotransmitter'/exp OR 'neurotropic agent'/exp OR 'serotonin'/exp OR 'corticosteroid'/exp OR 'corticotropin'/exp OR 'brain derived neurotrophic factor'/exp OR 'transforming growth factor beta'/exp OR 'noradrenalin'/exp OR 'dopamine'/exp OR 'nerve growth factor'/exp OR 'blood sample':ab,ti OR 'endocrine':ab,ti OR 'neurological pathway':ab,ti OR 'neuroanatom*':ab,ti OR 'scientific basis':ab,ti OR '5-ht':ab,ti OR 'bdnf':ab,ti OR 'hpa axis':ab,ti OR 'monoamine':ab,ti	2,180,125
#5	#1 AND #2 AND #3 AND #4	387

CENTRAL

	Searches	Results
#1	MeSH descriptor: [Depression] explode all trees	14182
#2	MeSH descriptor: [Depressive Disorder] explode all trees	13423
#3	depression:ti,ab,kw	89097

#4	depressive disorder:ti,ab,kw	21805
#5	depressive	32983
#6	depress$	522
#7	dysthymia	966
#8	#1 OR #2 OR #3 OR #4 OR #5 OR #6 OR #7	95773
#9	MeSH descriptor: [Plants, Medicinal] explode all trees	953
#10	MeSH descriptor: [Drugs, Chinese Herbal] explode all trees	3808
#11	MeSH descriptor: [Medicine, Chinese Traditional] explode all trees	1276
#12	MeSH descriptor: [Medicine, Kampo] explode all trees	48
#13	MeSH descriptor: [Medicine, Korean Traditional] explode all trees	34
#14	MeSH descriptor: [Herbal Medicine] explode all trees	68
#15	MeSH descriptor: [Prescription Drugs] explode all trees	113
#16	traditional Korean medicine:ti,ab,kw	144
#17	traditional Chinese medicine:ti,ab,kw	8714
#18	Traditional oriental medicine:ti,ab,kw	41
#19	Kampo medicine:ti,ab,kw	203
#20	alternative medicine:ti,ab,kw	4370
#21	complementary medicine:ti,ab,kw	2090
#22	herb*:ti,ab,kw	12932
#23	decoction*:ti,ab,kw	4081
#24	botanic*:ti,ab,kw	450
#25	#9 OR #10 OR #11 OR #12 OR #13 OR #14 OR #15 OR #16 OR #17 OR #18 OR #19 OR #20 OR #21 OR #22 OR #23 OR #24	27859
#26	MeSH descriptor: [Neurosciences] explode all trees	78
#27	MeSH descriptor: [Serotonin] explode all trees	1119
#28	MeSH descriptor: [Adrenal Cortex Hormones] explode all trees	15283
#29	MeSH descriptor: [Adrenocorticotropic Hormone] explode all trees	1473
#30	MeSH descriptor: [Brain-Derived Neurotrophic Factor] explode all trees	376
#31	MeSH descriptor: [Transforming Growth Factor beta] explode all trees	461
#32	MeSH descriptor: [Norepinephrine] explode all trees	2916
#33	MeSH descriptor: [Dopamine] explode all trees	1348
#34	MeSH descriptor: [Nerve Growth Factor] explode all trees	96
#35	MeSH descriptor: [Endocrine System] explode all trees	4572
#36	MeSH descriptor: [Neurotransmitter Agents] explode all trees	23832
#37	(blood sample):ti,ab,kw	22091
#38	(neurological pathway):ti,ab,kw	151
#39	(neuroanatom*):ti,ab,kw	386
#40	(neurotropic factor):ti,ab,kw	66
#41	(scientific basis):ti,ab,kw	1026
#42	(5HT):ti,ab,kw	246
#43	(HPA axis):ti,ab,kw	1133
#44	(BDNF):ti,ab,kw	1607
#45	(monoamine):ti,ab,kw	1465
#46	#26 OR #27 OR #28 OR #29 OR #30 OR #31 OR #32 OR #33 OR #34 OR #35 OR #36 OR #37 OR #38 OR #39 OR #40 OR #41 OR #42 OR #43 OR #44 OR #45	72130
#47	#8 AND #25 AND #46	111

AMED via EBSCO

	Searches	Results
#1	SU 'depressive disorder' OR SU depression OR TX depressive OR TX depression	8,678
#2	SU 'plants, medicinal' OR SU 'Drugs, Chinese Herbal' OR SU 'Medicine, Chinese Traditional' OR SU 'Medicine, Kampo' OR SU 'Medicine, Korean Traditional' OR SU 'Herbal Medicine' OR SU 'Prescription Drugs' OR TX traditional Korean medicine OR TX traditional Chinese medicine OR TX traditional oriental medicine OR TX Kampo medicine OR TX alternative medicine OR TX 'complementary medicine' OR TX herb* OR TX decoction* OR TX botanic*	42,910

#3	TX neuroscience OR TX 'blood sample' OR TX endocrine OR TX 'neurological pathway' OR TX neuroanatom* OR TX neurotransmitter OR TX 'neurotropic factor' OR TX 'scientific basis' OR TX '5-HT' OR TX serotonin OR TX cortisol OR TX corticostero* OR TX ACTH OR TX HPA axis OR TX BDNF OR TX monoamine OR TX 'transforming growth factor' OR TX norepinephrine OR TX dopamine OR TX nerve growth factor	4,529
#4	#1 AND #2 AND #3	120

PsycARTICLES via ProQuest

	Searches	Results
#1	MJMAINSUBJECT.EXACT("Depression (Emotion)") OR SU(('dysthymic disorder') OR depressive* OR depress* OR dysthymia*) OR AB(('dysthymic disorder') OR depressive* OR depress* OR dysthymia*) OR TI(('dysthymic disorder') OR depressive* OR depress* OR dysthymia*)	13,476
#2	MJMAINSUBJECT.EXACT("Medicinal Herbs and Plants") OR MJMAINSUBJECT.EXACT("Alternative Medicine") OR SU('traditional Korean medicine' OR 'traditional Chinese medicine' OR 'Traditional oriental medicine' OR 'Kampo medicine' OR 'alternative medicine' OR 'complementary medicine' OR 'herb*' OR 'decoction*' OR 'botanic*') OR SU('traditional Korean medicine' OR 'traditional Chinese medicine' OR 'Traditional oriental medicine' OR 'Kampo medicine' OR 'alternative medicine' OR 'complementary medicine' OR 'herb*' OR 'decoction*' OR 'botanic*') OR AB('traditional Korean medicine' OR 'traditional Chinese medicine' OR 'Traditional oriental medicine' OR 'Kampo medicine' OR 'alternative medicine' OR 'complementary medicine' OR 'herb*' OR 'decoction*' OR 'botanic*') OR TI('traditional Korean medicine' OR 'traditional Chinese medicine' OR 'Traditional oriental medicine' OR 'Kampo medicine' OR 'alternative medicine' OR 'complementary medicine' OR 'herb*' OR 'decoction*' OR 'botanic*')	362
#3	'randomized controlled trial' OR 'controlled clinical trial' OR randomized OR placebo OR randomly OR trial OR groups	153,205
#4	(MJMAINSUBJECT.EXACT("Neurosciences") OR MJMAINSUBJECT.EXACT("Endocrine System") OR MJMAINSUBJECT.EXACT("Neurotrophic Factor") OR MJMAINSUBJECT.EXACT("Neurotransmitters") OR MJMAINSUBJECT.EXACT("Serotonin") OR MJMAINSUBJECT.EXACT("Adrenal Cortex Hormones") OR MJMAINSUBJECT.EXACT("Brain Derived Neurotrophic Factor") OR MJMAINSUBJECT.EXACT("Norepinephrine") OR MJMAINSUBJECT.EXACT("Dopamine") OR MJMAINSUBJECT.EXACT("Nerve Growth Factor")) OR SU('neurosciences' OR 'endocrine' OR 'neurotransmitter' OR 'blood sample' OR 'neurological pathway' OR neuroanatom* OR 'scientific basis' OR '5-ht' OR bdnf OR monoamine) OR AB('neurosciences' OR 'endocrine' OR 'neurotransmitter' OR 'blood sample' OR 'neurological pathway' OR neuroanatom* OR 'scientific basis' OR '5-ht' OR bdnf OR monoamine) OR TI('neurosciences' OR 'endocrine' OR 'neurotransmitter' OR 'blood sample' OR 'neurological pathway' OR neuroanatom* OR 'scientific basis' OR '5-ht' OR bdnf OR monoamine)	6,081
	#1 AND #2 AND #3 AND #4	0

OASIS

	Searches	Results
#1	우울 AND 한약	4

KCI

	Searches	Results
#1	우울 AND 한약	19

CNKI

	Searches	Results
#1	SU=抑郁证 OR SU=忧郁症 OR SU=郁证 OR SU=脏躁 OR SU=depression OR SU='major depression' OR SU=Melancholia OR SU='involutional depression'	**789,297**
#2	SU=中药 OR SU=汤 OR SU=散 OR SU=丸 OR SU=方 OR SU=颗粒 OR SU=胶囊 OR SU=自拟 OR SU='Herbal medicine' OR SU='Chinese medicine' OR SU='Traditional chinese medicine' OR SU=Powder OR SU=Pill OR SU=Prescriptions OR SU=Capsule OR SU=Herb OR SU=Decoction	**2,526,351**
#3	SU=随机 OR SU=对照 OR SU=randomized OR SU=临床研究	**1,611,554**
#4	SU=神经科学 OR SU=血液样本 OR SU=内分泌 OR SU=神经通路 OR SU=神经解剖学 OR SU=神经递质 OR SU=神经营养因子 OR SU=科学依据 OR SU=5-羟色胺 SU=血清素 OR SU=皮质醇 OR SU='HPA 轴' OR SU=神经营养因子 OR SU=促肾上腺皮质激素 OR SU=转化生长因子 β OR SU=去甲肾上腺素 OR SU=多巴胺 OR SU=神经生长因子 OR SU=neuroscience OR SU='blood sample' OR SU=endocrine OR SU='neurological pathway' OR SU=neurotransmitter OR SU='neurotropic factor' OR SU='scientific basis' OR SU='5-HT' OR SU=serotonin OR SU=cortisol OR SU=ACTH OR SU='HPA axis' OR SU=BDNF OR SU=monoamine OR SU='TGF β' OR SU='transforming growth factor β' OR SU=norepinephrine OR SU=dopamine OR SU=NGF OR SU='nerve growth factor'	**545,063**
#5	#1 AND #2 AND #3 AND #4	**2071**

CiNii

	Searches	Results
#1	depressive disorder OR depression OR depressive OR depression OR 憂鬱症 OR 鬱病 OR 抑鬱 OR うつ病 OR 抑うつ OR エムディーディー OR だいうつびょうせいしょうがい OR ゆううつしょう OR デプレッション OR ディプレッション	**117,692**
#2	chinese medicine OR herbal medicine OR kampo OR 東洋医学 OR 漢方 OR 湯 OR 散 OR 丸 OR 方 OR 飲 OR 煎 OR 顆粒	**3,551,150**
#3	randomized OR randomised OR ランダム化 OR 無作為化	**225,560**
#4	neuroscience OR 'blood sample' OR endocrine OR 'neurological pathway' OR neuroanatom* OR neurotransmitter OR 'neurotropic factor' OR 'scientific basis' OR '5-ht' OR serotonin OR cortisol OR corticostero* OR acth OR 'hpa axis' OR bdnf OR monoamine OR 'tgf β' OR 'transforming growth factor β' OR ne OR norepinephrine OR da OR dopamine OR ngf OR 'nerve growth factor' OR 神経科学 OR 血液サンプル OR 内分泌 OR 神経経路 OR 神経伝達物質 OR 神経栄養因子 OR 科学的根拠 OR セロトニン OR コルチゾール OR モノアミン OR トランスフォーミング成長因子 β OR ノルエピネフリン OR ドーパミン OR 神経成長因子	**1,157,377**
#5	#1 AND #2 AND #3 AND #4	**10**
