# Supplementary material for: Neuroendocrine Biomarkers of Herbal Medicine for Major Depressive Disorder: A Systematic Review and Meta-Analysis
Source: Pharmaceuticals (Basel). 2023 Aug 18;16(8):1176. doi: 10.3390/ph16081176 (PMC10458856; doi:10.3390/ph16081176)
Supplement: Supplementary file 1 [file pharmaceuticals-16-01176-s001.zip › Supplementary table S1. Characteristics of included study.pdf]

Supplementary Table S1. Characteristics of Included Study

First Author (year)	Sample size (male/female)	Mean age (years)	Diagnostic tool (severity criteria for inclusion)	Treatment intervention‡ (A)	Control intervention (B)	Duration (F/U)	Outcome measurements and results
Li (26) (2016)	(A) 40 (22/18)<br>(B) 40 (19/21)	(A) 28.72±8.79<br>(B) 27.78±8.56	CCMD-3 (HAMD ≥8)	Chaifu Jieyu prescription bid & Mirtazapine 10–30 mg qd	Mirtazapine 10–30 mg qd	8 weeks	① 5-HT: (A)>(B)*<br>② NE: (A)>(B)*<br>③ HAMD: (A)>(B)*
Wu (27) (2020)	(A) 40 (20/20)<br>(B) 40 (20/20)	(A) 38.96±5.36<br>(B) 40.41±1.97	CCMD-3	Chaihu Longgu Muli decoction 200 mL bid & Paroxetine 30 mg qd	Paroxetine 30 mg qd	4 weeks	① 5-HT: (A)>(B)*<br>② CORT: (A)>(B)*<br>③ HAMD: (A)>(B)*<br>④ SDS: (A)>(B)*
Zhu (28) (2020)	(A) 42 (18/24)<br>(B) 41 (12/29)	(A) 52.17±12.69<br>(B) 48.98±12.21	CCMD-3 & DSM5 (35≥ HAMD 24≥20)	Chaihu Longgu Muli decoction 150 mL bid & Escitalopram 10 mg bid	Escitalopram 10 mg bid	8 weeks	① BDNF: (A)>(B)*<br>② HAMD: (A)>(B)*<br>③ HAMD ER: (A)>(B)*
Liu (29) (2017)	(A) 51 (19/32)<br>(B) 51 (20/31)	(A) 39.67±5.60<br>(B) 38.91±5.78	CCMD-3	Chaihu Shugan powder 300 mL & Venlafaxine 75–150 mg qd	Venlafaxine 75–150 mg qd	8 weeks	① 5-HT: (A)>(B)*<br>② HAMD: (A)>(B)*<br>③ HAMD ER: (A)>(B)*
Pan (30) (2018)	(A) 50 (35/15)<br>(B) 50 (37/13)	(A) 40.19±4.52<br>(B) 40.53±4.60	CCMD-3 (HAMD 17≥17)	Chaihu Shugan powder 150 mL bid & Paroxetine 20 mg qd	Paroxetine 20 mg qd	4 weeks	① 5-HT: (A)>(B)*<br>② HAMD ER: (A)>(B)*
Sun (31) (2020)	(A) 48 (21/27)<br>(B) 48 (22/26)	(A) 39.86±7.74<br>(B) 39.63±7.51	CCMD-3 (HAMD 17≥17)	Chaihu Shugan powder 150 mL bid & Escitalopram 20 mg qd	Escitalopram 20 mg qd	8 weeks	① 5-HT: (A)>(B)*<br>② NE: (A)>(B)*<br>③ HAMD: (A)>(B)*<br>④ HAMD ER: (A)>(B)*
Tan (32) (2020)	(A) 43 (21/22)<br>(B) 43 (20/23)	(A) 48.45±10.61<br>(B) 49.16±10.13	CCMD-3	Chaihu Shugan powder 250 mL bid & Doxepin 25–50 mg bid	Doxepin 25–50 mg bid	8 weeks	① 5-HT: (A)>(B)*<br>② HAMD: (A)>(B)*<br>③ SDS: (A)>(B)*

First Author (year)	Sample size (male/female)	Mean age (years)	Diagnostic tool (severity criteria for inclusion)	Treatment intervention‡ (A)	Control intervention (B)	Duration (F/U)	Outcome measurements and results
Li (23) (2007)	(A) 32 (15/17)<br>(B) 31 (14/17)	(A) 39.7±11.9<br>(B) 38.6±13.6	CCMD-3 & ICD-10 (HAMD 17≥20)	Danzhi Xiaoyao powder 12 g bid & placebo Maprotiline	Maprotiline 25–125 mg qd (1st week) avg. 200 mg max 250 mg qd (2nd–6th week) & placebo Danchisoyo-san	6 weeks	① 5-HT: NS<br>② BDNF: NS<br>③ CORT: (A)>(B)*<br>④ HAMD: NS
Zhou (33) (2015)	(A) 40 (15/25)<br>(B) 40 (18/22)	(A) 36±5.5<br>(B) 36±5.3	CCMD-3 (35≥ HAMD 24≥18)	Fuyang Shugan Juanpi prescription 200 mL tid	Fluoxetine 20 mg qd	6 weeks	① 5-HT: NS<br>② HAMD: NS
Liang (34) (2012)	(A) 30 (23/7)<br>(B) 30 (22/8)	(A) 38.00±11.47<br>(B) 38.72±12.38	CCMD-3 (HAMD 17≥17)	Guipi decoction 200 mL bid	Fluoxetine 20 mg qd	6 weeks	① 5-HT: NS<br>② HAMD: NS<br>③ HAMD ER: NS<br>④ MADRS: (A)>(B)*
Qin (35) (2020)	(A) 46<br>(B) 46	NR	ICD-10 (35≥ HAMD ≥20)	Jiawei Chaihu decoction 200 mL bid & Paroxetine 20 mg qd	Paroxetine 20 mg qd	3 weeks	① 5-HT: (A)>(B)*<br>② 5-HIAA: (A)>(B)*<br>③ NE: (A)>(B)*<br>④ BDNF: (A)>(B)*<br>⑤ NGF: (A)>(B)*<br>⑥ HAMD: (A)>(B)*
Liu (36) (2021)	(A) 48 (15/33)<br>(B) 48 (12/36)	(A) 31.75±7.61<br>(B) 31.63±7.50	CCMD-3	Jiawei Chaihu decoction 200 mL bid & Paroxetine 20–50 mg qd	Paroxetine 20–50 mg qd	24 weeks	① 5-HT: (A)>(B)*<br>② 5-HIAA: (A)>(B)*<br>③ NE: (A)>(B)*<br>④ BDNF: (A)>(B)*<br>⑤ NGF: (A)>(B)*<br>⑥ HAMD RETARDATION: (A)>(B)*<br>⑦ HAMD INSOMNIA: (A)>(B)*<br>⑧ HAMD SOMATIC ANXIETY: (A)>(B)*<br>⑨HAMD PSYCHIC ANXIETY : (A)>(B)*<br>⑩ HAMD DEPRESSED MOOD: (A)>(B)*

First Author (year)	Sample size (male/female)	Mean age (years)	Diagnostic tool (severity criteria for inclusion)	Treatment intervention‡ (A)	Control intervention (B)	Duration (F/U)	Outcome measurements and results
Tong (37) (2016)	(A) 30 (18/12)<br>(B) 30 (17/13)	(A) 35.1±2.5<br>(B) 34.6±2.1	ICD-10	Jiawei Xiaoyao powder bid	Paroxetine<br>20 mg qd (1st week)<br>30 mg qd (2nd week)<br>40 mg qd (3rd week)<br>& placebo Gamisoyo-san bid	8 weeks	① BDNF: (A)>(B)*<br>② HAMD: (A)>(B)*<br>③ HAMD ER: (A)>(B)*
Tong (38) (2018)	(A) 58<br>(B) 58	(A) 37.42±3.75<br>(B) 37.11±3.39	CCMD-3 (HAMD >17)	Jiawei Xiaoyao powder bid & Fluoxetine 20 mg qd	Fluoxetine 20 mg qd	8 weeks	① BDNF: (A)>(B)*<br>② HAMD: (A)>(B)*<br>③ HAMD ER: (A)>(B)*
Wang (39) (2022)	(A) 41 (17/24)<br>(B) 41 (16/25)	(A) 35.28±4.42<br>(B) 35.21±4.36	ICD-10 (HAMD 17>17)	Jiawei Xiaoyao powder 150 mL bid & Fluoxetine 20 mg qd	Fluoxetine 20 mg qd	8 weeks	① BDNF: (A)>(B)*<br>② HAMD: (A)>(B)*<br>③ HAMD ER: (A)>(B)*<br>④ TESS: (A)>(B)*
Huo (40) (2013)	(A) 43 (18/25)<br>(B) 42 (17/25)	(A) 41.2±7.35<br>(B) 42.3±7.52	CCMD-3 (35≥ HAMD ≥7)	Jieyu Anshen decoction bid & Paroxetine 20–50 mg qd	Paroxetine 20–50 mg qd	6 weeks	① BDNF: (A)>(B)*<br>② HAMD: (A)>(B)*<br>③ HAMD ER: (A)>(B)*
Guo (41) (2015)	(A) 49 (19/30)<br>(B) 49 (20/29)	(A) 47.11±8.65<br>(B) 46.85±8.71	CCMD-3 (35≥ HAMD ≥7)	Jieyu Anshen Dingzhi decoction 200 mL bid & Paroxetine 20–50 mg qd	Paroxetine 20–50 mg qd	12 weeks	① BDNF: (A)>(B)*<br>② HAMD: (A)>(B)*
Wang (42) (2020)	(A) 46 (22/24)<br>(B) 46 (23/23)	(A) 40.4±8.8<br>(B) 42.1±9.2	ICD-10 (HAMD ≥17)	Jieyu pill 4 g tid & Agomelatine 25–50 mg qd	Agomelatine 25–50 mg qd	8 weeks	① BDNF: (A)>(B)*<br>② HAMD: (A)>(B)*<br>③ TESS: NS
Li (43) (2017)	(A) 30 (12/18)<br>(B) 30 (11/19)	(A) 37.20±2.3<br>(B) 38.00±2.6	DSM-5 (HAMD 24≥20)	Jinkui Shenqi pill 200 mL bid & Escitalopram 10–20 mg qd	Escitalopram 10–20 mg qd	6 weeks	① NGF: (A)>(B)*<br>② HAMD: (A)>(B)*<br>③ HAMD ER: (A)>(B)*
Wang (44) (2018)	(A) 66 (43/23)<br>(B) 65 (40/25)	(A) 39.86±7.36<br>(B) 40.61±6.97	CCMD-3	Jiuwei Zhenxing granules 1 sachet (6 g) tid & Duloxetine 60 mg qd	Duloxetine 60 mg qd	6 weeks	① 5-HT: (A)>(B)*<br>② CORT: (A)>(B)*<br>③ HAMD: (A)>(B)*<br>④ SDS: (A)>(B)*

First Author (year)	Sample size (male/female)	Mean age (years)	Diagnostic tool (severity criteria for inclusion)	Treatment intervention‡ (A)	Control intervention (B)	Duration (F/U)	Outcome measurements and results
Wang (45) (2017)	(A) 100 (58/42)<br>(B) 100 (61/39)	(A) 43.25±5.41<br>(B) 44.37±6.10	CCMD-3	Self-made herbal medicine 150 mL bid & Venlafaxine 75–150 mg qd	Venlafaxine 75–150 mg qd	8 weeks	① 5-HT: (A)>(B)*<br>② DA: (A)>(B)*<br>③ NE: (A)>(B)*<br>④ HAMD: (A)>(B)*<br>⑤ HAMD ER: (A)>(B)*
Cheng (46) (2018)	(A) 50<br>(B) 50	NR	CCMD-3	Self-made herbal medicine 150 mL bid & Sertraline 50–200 mg qd	Sertraline 50–200 mg qd	12 weeks	① BDNF: (A)>(B)*<br>② HAMD: (A)>(B)*
Du (47) (2018)	(A) 78 (38/40)<br>(B) 78 (34/44)	(A) 33.78±9.84<br>(B) 34.25±9.96	ICD-10	Self-made herbal medicine 150 mL bid & Citalopram 10 mg qd (1st week) 20 mg qd (2nd week–)	Citalopram 10 mg qd (1st week) 20 mg qd (2nd week–)	6 weeks	① 5-HT: (A)<(B)*<br>② NE: (A)>(B)*<br>③ CORT: (A)>(B)*<br>④ HAMD: (A)>(B)*<br>⑤ HAMD ER: (A)>(B)*<br>⑥ MADRS: (A)>(B)*
Gong (48) (2016)	(A) 70 (37/33)<br>(B) 70 (39/31)	(A) 46.9±10.8<br>(B) 46.3±10.4	CCMD-3	Shugan Jieyu capsules (3 pills) & Duloxetine 30–60 mg qd	Duloxetine 30–60 mg qd	6 weeks	① BDNF: (A)>(B)*<br>② HAMD: (A)>(B)*
Chen (49) (2021)	(A) 150 (69/81)<br>(B) 150 (72/78)	(A) 42.19±11.84<br>(B) 41.93±12.93	CCMD-3 (HAMD ≥18)	Shugan Jieyu capsules (3 pills) & Venlafaxine 75 mg qd (1st week) 150 mg qd (2nd week–)	Venlafaxine 75 mg qd (1st week) 150 mg qd (2nd week–)	8 weeks	① 5-HT: (A)>(B)*<br>② DA: (A)>(B)*<br>③ NE: (A)>(B)*<br>④ HAMD ER: (A)>(B)*
Wu (50) (2019)a	(A) 40 (20/20)<br>(B) 40 (20/20)	(A) 38.96±5.36<br>(B) 40.41±1.97	ICD-10	Sini powder 100 mL bid & Paroxetine 20 mg qd	Paroxetine 20 mg qd	8 weeks	① 5-HT: (A)>(B)*<br>② HAMD: (A)>(B)*<br>③ SDS: (A)>(B)*
Wu (51) (2019)b	(A) 40 (20/20)<br>(B) 40 (20/20)	(A) 38.96±5.36<br>(B) 40.41±1.97	ICD-10	Sini powder 100 mL bid & Mirtazapine 30 mg qd	Mirtazapine 30 mg qd	8 weeks	① 5-HT: (A)>(B)*<br>② HAMD: (A)>(B)*<br>③ SDS: (A)>(B)*
Wang (52) (2005)	(A) 32 (15/17)<br>(B) 31 (10/21)	(A) 34.47±12.67<br>(B) 30.48±10.85	CCMD-3	Wangyou decoction 100 mL bid	Fluoxetine 20 mg qd	4 weeks	① 5-HIAA: NS<br>② HAMD: NS<br>③ HAMD ER: NS<br>④ MADRS: (A)>(B)*

First Author (year)	Sample size (male/female)	Mean age (years)	Diagnostic tool (severity criteria for inclusion)	Treatment intervention‡ (A)	Control intervention (B)	Duration (F/U)	Outcome measurements and results
Su (53) (2020)	(A) 67 (40/27)<br>(B) 67 (42/25)	(A) 43.5±3.9<br>(B) 43.9±3.7	CCMD-3	Xiaochaihu decoction 150 mL bid	Paroxetine or Fluoxetine 20 mg qd	6 weeks	① 5-HT: (A)>(B)*<br>② NF: (A)>(B)*<br>③ HAMD ER: (A)>(B)*
Bo (54) (2022)	(A) 53 (24/29)<br>(B) 53 (25/28)	(A) 40.12±5.12<br>(B) 40.61±5.36	DSM-IV	Xiaoyao pill 9 g bid & Venlafaxine 25–225 mg qd	Venlafaxine 25–225 mg qd	8 weeks	① 5-HT: (A)>(B)*<br>② BDI: (A)>(B)*<br>③ BDI ER: (A)>(B)*
Zhu (55) (2016)	(A) 13 (6/7)<br>(B) 9 (2/7)	(A) 42.07±12.5<br>(B) 44.89±14.98	CCMD-3 (HAMD 24>20)	Yueju Wan 23 g qd & Fluoxetine 20 mg qd	Fluoxetine 20 mg qd & placebo Wolguk-hwan 23 g qd	1 week	① BDNF: NS<br>② HAMD: (A)>(B)*<br>③ SDS: NS
	(A) 10 (1/9)<br>(B) 9 (2/7)	(A) 52.7±9.7<br>(B) 44.89±14.98	CCMD-3 (HAMD 24>20)	Yueju Wan 18 g qd & Fluoxetine 20 mg qd	Fluoxetine 20 mg qd & placebo Wolguk-hwan 23 g qd	1 week	① BDNF: NS<br>② HAMD: NS<br>③ SDS: NS

Note: '*' indicates a significant difference, p<0.05. 'NS' indicates no significant difference, p>0.05. 'NR' = not reported. (A) = Treatment intervention; (B) = Control intervention.

5-HIAA = 5-hydroxyindoleacetic acid; 5-HT = 5-hydroxytryptamine (Serotonin); BDI = Beck Depression Inventory; BDNF = Brain Derived Neurotrophic Factor; CCMD = Chinese Classification of Mental Disorders; DSM = Diagnostic Statistical Manual; CORT = Cortisol; DA = Dopamine; ER = Effective Rate; HAMD = Hamilton Depression Scale; ICD = International Statistical Classification of Disease; MADRS = Montgomery–Åsberg Depression Rating Scale; NE = Norepinephrine; NF = Neurotrophic Factor; NGF = Nerve growth factor; SDS = Self-Rating Depression Scale; TESS = Treatment-Emergent Signs and Symptom
