# Supplementary material for: Neuroendocrine Biomarkers of Herbal Medicine for Major Depressive Disorder: A Systematic Review and Meta-Analysis
Source: Pharmaceuticals (Basel). 2023 Aug 18;16(8):1176. doi: 10.3390/ph16081176 (PMC10458856; doi:10.3390/ph16081176)
Supplement: Supplementary file 1 [file pharmaceuticals-16-01176-s001.zip › Supplementary table S2. Results of sensitivity analysis.pdf]

Supplementary Table S2. Results of sensitivity analysis after removal of studies in which confidence interval does not overlap with the confidence interval of the pooled effect.

**Outcomes**	**RCT**	**Sample size**	**RR or SMD**	**95% CI**	**I^2 value**	**Z value**	**P value**
**HM combined with antidepressants vs. antidepressants alone**							
5-HT	9	1152	SMD 1.26	0.87, 1.66	89%	6.29	< 0.0001
NE	6	954	SMD 1.20	0.84, 1.56	84%	6.51	< 0.0001
BDNF	9	798	SMD 1.11	0.84, 1.39	69%	7.93	< 0.0001
CORT	4	224	SMD -3.08	-3.48,-2.67	52%	14.80	< 0.00001
HAMD	12	1183	SMD -1.45	-1.75, -1.14	81%	9.33	< 0.00001

5-HT = 5-hydroxytryptamine (Serotonin); BDNF = Brain Derived Neurotrophic Factor; CI = Confidence Interval; CORT = Cortisol; HAMD = Hamilton Depression Scale; NE = Norepinephrine; RCT = Randomized Controlled Trials; RR = Risk Ratio; SMD = Standardized Mean Difference
